# Lecanemab Reduces Neuropsychiatric Symptoms and Related Regional Brain Amyloid Load in Early Alzheimer's Disease: A Preliminary Prospective Study

**DOI:** 10.1002/cns.70974

**Published:** 2026-06-11

**Authors:** Yan Chang, Hua Li, Xiaojie Liu, Xuemei Li, Jiajin Liu, Jingbin Song, Huaping Fu, Xiaodan Xu, Yuan Wang, Qianyao Wang, Na Ren, Jilin Chen, Xin Deng, Xue Zhang, Luofei Zuo, Bo Zhou, Xuan Sun, Zihan Li, Yuanyan Cao, Runze Wu, Jianjun Jia, Hairong Qian, Ruimin Wang

**Affiliations:** ^1^ Department of Nuclear Medicine, the First Medical Center Chinese PLA General Hospital Beijing China; ^2^ Senior Department of Neurology Chinese PLA General Hospital Beijing China; ^3^ Graduate School of the Chinese PLA General Hospital Beijing China; ^4^ Department of Cadres' Outpatient The First Medical Center of Chinese PLA General Hospital Beijing China; ^5^ Department of Neurology, the Second Medical Center & National Clinical Research Center for Geriatric Diseases Chinese PLA General Hospital Beijing China; ^6^ Beijing United Imaging Healthcare Beijing China; ^7^ Institute of Geriatrics, the Second Medical Center & National Clinical Research Center of Geriatric Diseases Chinese PLA General Hospital Beijing China

**Keywords:** Alzheimer's disease, amyloid beta positron emission tomography, lecanemab, MRI, neuropsychiatric symptoms

## Abstract

**Aim:**

This prospective study examined whether lecanemab was associated with changes in neuropsychiatric symptoms (NPS) and investigated their associations with cerebral amyloid burden in patients with early‐stage Alzheimer's disease (AD).

**Methods:**

Fourteen eligible participants underwent amyloid positron emission tomography, magnetic resonance imaging, and neuropsychological assessments at baseline and following 6 months of lecanemab treatment. Neuropsychological assessments included the Clinical Dementia Rating, Mini‐Mental State Examination (MMSE), Montreal Cognitive Assessment (MoCA), Hamilton Depression Rating Scale, Hamilton Anxiety Scale (HAMA), and Neuropsychiatric Inventory (NPI).

**Results:**

MMSE and MoCA remained stable, while amyloid burden decreased after 6 months of treatment (*p* < 0.05). HAMA, total NPI score, and NPI sub‐scores for psychosis, hyperactivity, and apathy were also decreased (*p* < 0.05). Improvements in NPS were associated with lower amyloid burden in the hippocampus, amygdala, thalamus, inferior frontal gyrus (IFG), and anterior cingulate gyrus. These clinical improvements were associated with increased fractal dimension in the middle cingulate cortex and decreased sulcal depth in the IFG.

**Conclusions:**

These findings suggest that, in early AD, lecanemab treatment may be associated with benefits beyond cognitive stabilization, including possible improvement in NPS, which may relate to amyloid clearance and structural changes in relevant brain regions.

## Graphical abstract

This prospective study shows that 6 months of lecanemab treatment significantly reduces brain amyloid load and improves neuropsychiatric symptoms in early Alzheimer's disease. These improvements are linked to amyloid clearance and structural changes in emotion‐related brain regions, indicating benefits beyond cognition.

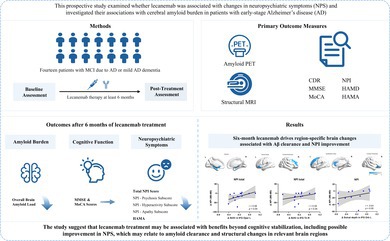

## 1 Introduction

Alzheimer's disease (AD), the primary etiology of dementia, is neuropathologically characterized by the accumulation of amyloid‐beta (Aβ) plaques and neurofibrillary tau tangles [1]. Although cognitive decline is a hallmark feature, neuropsychiatric symptoms (NPS), including apathy, agitation, depression, anxiety, and psychosis, are increasingly recognized as prevalent across the AD continuum [2, 3]. In a cross‐sectional cohort with Aβ deposition, at least one NPS was reported in 28.0% of cognitively unimpaired individuals with subjective cognitive decline, 40.5% of those with mild cognitive impairment (MCI), and 81.0% of those with dementia [4]. In patients with AD, NPS have been associated with faster progression to dementia, greater functional decline, diminished quality of life, and higher mortality risk [5]. Furthermore, NPS impose a substantial burden on caregivers, significantly compromising their psychological well‐being and overall quality of life [5].

Even when identified, NPS are frequently managed with off‐label psychotropic drugs, which often exhibit limited efficacy and may potentially have unfavorable safety profiles [5]. Therefore, improving NPS management and discovering new therapeutic targets remain important clinical and research priorities. A post hoc analysis of the 10‐item Neuropsychiatric Inventory Questionnaire (NPI‐10) in the EMERGE trial reported drug‐placebo differences favoring aducanumab for agitation/aggression, apathy/indifference, depression/dysphoria, aberrant motor behavior, and delusions [6].

Aβ deposition, assessed with cerebrospinal fluid (CSF) biomarkers and positron emission tomography (PET) imaging, has been linked to specific NPS, including agitation, apathy, and depression [7, 8]. Several studies have also reported positive correlations between NPS severity and both Aβ and Tau pathological burden measured by CSF biomarkers or PET imaging [8, 9]. Neuroimaging studies further suggest that the presence and severity of NPS may be associated with PET‐derived Aβ burden, quantified by standardized uptake value ratio (SUVr) or the Centiloid (CL) scale, in aging and early cognitive impairment, supporting a shared pathophysiological substrate [10, 11, 12, 13]. Magnetic resonance imaging (MRI) also contributes to the assessment of structural brain changes related to NPS in AD [14]. However, the relationship between NPS severity and regional Aβ burden, particularly within NPS‐relevant brain regions, is not well understood.

Lecanemab is a humanized monoclonal antibody that selectively targets soluble Aβ protofibrils. In early‐stage AD, it has demonstrated efficacy in reducing amyloid plaque burden and attenuating cognitive and functional decline across validated clinical endpoints [15], which supported its regulatory approval. However, the effect of anti‐amyloid therapy on NPS trajectories, particularly during the early treatment phase, is still unknown. Given the substantial impact of NPS on patients and caregivers, and the proposed link between Aβ pathology and specific NPS domains, determining whether lecanemab is associated with improvement in these symptoms is clinically relevant.

In this prospective longitudinal study, we employed Aβ PET to quantify 6‐month changes in Aβ burden and examined their associations with NPS trajectories during lecanemab treatment in early‐stage AD. Specifically, we explored whether the magnitude of NPS improvement correlated with regional reductions in amyloid deposition.

## 2 Methods

### 2.1 Study Participants

This prospective longitudinal observational study enrolled participants aged 50–90 years who met criteria for MCI due to AD or mild AD dementia (early‐stage AD, clinical stages 3–4) according to the 2024 Alzheimer's Association revised criteria [1]. Amyloid positivity on PET was adjudicated via visual interpretation by two nuclear medicine experts, following the Japanese Alzheimer's Disease Neuroimaging Initiative (J‐ADNI) PET Core criteria [16]. All participants were deemed eligible for lecanemab treatment by a clinical expert panel and completed at least 6 months of treatment (10 mg/kg every 2 weeks). Participants were maintained on stable doses of cholinesterase inhibitors or N‐methyl‐D‐aspartic acid receptor antagonists before enrollment, and these doses remained unchanged during the 6‐month treatment period to minimize pharmacological confounding. No participant was receiving psychotropic medication or hypnotics. Based on the Clarity AD study and the appropriate‐use recommendations for lecanemab [15, 17], participants were excluded if baseline brain MRI revealed any of the following: more than 5 microhemorrhages (≤ 10 mm in greatest diameter), a single macrohemorrhage > 10 mm, superficial siderosis, vasogenic edema, cerebral contusion, encephalomalacia, aneurysms, vascular malformations, infective lesions, multiple lacunar infarcts or stroke in major vascular territory, severe small‐vessel disease or white matter lesions (Fazekas score ≥ 3), space‐occupying lesions or brain tumors, or an inadequately controlled bleeding disorder (including platelet count < 50,000 or international normalized ratio > 1.5 in participants not receiving anticoagulants, e.g., warfarin) [15]. Written informed consent was obtained from all participants.

### 2.2 Neuropsychological Assessment

Comprehensive neuropsychological assessments were administered at baseline (M0, before the first lecanemab infusion) and at month 6 (M6), including the Clinical Dementia Rating (CDR), Mini‐Mental State Examination (MMSE), Montreal Cognitive Assessment (MoCA), Hamilton Depression Rating Scale (HAMD), Hamilton Anxiety Scale (HAMA), and Neuropsychiatric Inventory (NPI). NPI items were clustered into four subsyndromes: (1) hyperactivity (agitation, elation, disinhibition, irritability, and aberrant motor behavior); (2) psychosis (delusion, hallucination, and night‐time behavioral disturbance); (3) affective symptoms (anxiety and depression); (4) apathy (apathy and appetite) [18].

### 2.3 PET/CT Acquisitions

All participants underwent amyloid PET/CT with [^11^C] Pittsburgh Compound‐B (^11^C‐PIB) [11] at the Department of Nuclear Medicine, Chinese PLA General Hospital. The detailed protocol for image acquisition, reconstruction, processing, and ^11^C‐PIB quantification has been described previously [19].

### 2.4 PET Image Processing

All ^11^C‐PIB PET images were motion corrected and co‐registered to each participant's high‐resolution T1‐weighted MR images and then normalized to Montreal Neurological Institute (MNI) space using the transformation derived from the T1‐weighted MR images using FSL (FMRIB Software Library). Regions of interest (ROIs) were defined on the MNI template using the Automated Anatomical Labeling (AAL) atlas. Selected ROIs included the thalamus, hippocampus, para‐hippocampal gyrus, amygdala, frontal lobe, olfactory tubercle, and cingulate cortex. Mean SUVr values were extracted from each ROI to serve as quantitative measures of amyloid burden for subsequent statistical analyses. Cortical gray matter SUVr values were calculated using the cerebellar gray matter as the reference region. CL values were also calculated for each participant from the 40–60 min post‐injection frames (Level‐2 CL pipeline, see Supporting Information for details) [20].

### 2.5 MR Image Processing

Gray matter volumes and cortical surface metrics for each ROI were derived from T1‐weighted MR images processed as previously described [19]. ROIs included the thalamus, hippocampus, para‐hippocampal gyrus, amygdala, frontal lobe, olfactory tubercle, and cingulate gyrus. Cortical surface metrics, including gyrification index, fractal dimension, sulcal depth, and cortical thickness, were processed with the Computational Anatomy Toolbox [21].

### 2.6 Statistical Analysis

Statistical analyses were performed using SPSS 22.0 (IBM Corp.). Baseline characteristics were summarized descriptively. Age and years of education at M0 were reported as mean or median values, as appropriate. Comparisons between M0 and M6 were performed using paired *t*‐test or Wilcoxon signed‐rank test depending on the results of the Shapiro–Wilk test for normality. Pearson or Spearman correlation analyses were used to evaluate associations between changes in NPS scores and concurrent changes in neuroimaging measures. To account for multiple comparisons, *p* values were adjusted using the Benjamini‐Hochberg false discovery rate (FDR) correction. Statistical significance was set as *p* < 0.05.

## 3 Results

### 3.1 Demographic Characteristics

Of the 16 initially enrolled participants, 14 were included in the final analysis; two were excluded due to incomplete follow‐up. Age ranged from 53 to 81 years, with a median [interquartile range, IQR] of 71 [6.75] years. Of the participants, 57% were female, and 42.9% were apolipoprotein E (APOE) ε4 carriers. MMSE (*p* = 0.344) and MoCA (*p* = 0.343) scores did not change significantly between M0 and M6. CDR scores remained stable throughout the 6 months of lecanemab treatment period; 12 participants maintained a score of 0.5, while 2 retained a score of 1. No amyloid‐related imaging abnormalities (ARIA) were observed. M0 and M6 demographic characteristics and clinical outcomes are summarized in Table 1.

**TABLE 1:** Demographic and clinical characteristics of the study cohort.

	M0 (*n* = 14)	M6 (*n* = 14)	M6‐M0	*z* values	*p*	Effect size (*r*)
Age, year	71 (6.75)	—	—	—	—	
Education, year	12.5 (4.75)	—	—	—	—	
Female sex, *N* (%)	8 (57%)	—	—	—	—	
APOEε4 carrier, *N* (%)	6 (42.9%)	—	—	—	—	
CDR (0.5/1)	12/2	12/2	—	—	—	
MMSE	24.5 (5.25)	23.5 (6.25)	0 (2.75)	−1.044	0.344	0.28
MoCA	19.5 (8)	20 (5.75)	1 (3.25)	0.987	0.343	0.26
HAMD	5 (9)	3 (2)	−0.5 (6.5)	−1.194	0.25	0.32
HAMA	3 (5.25)	1 (2.75)	−1 (5)	−2.239	**0.025**	0.60
Total NPI score	22 (42.25)	8.5 (12.5)	−10.5 (25)	−2.667	**0.005**	0.71
Hyperactivity	6.5 (21.75)	4 (9.5)	−3.5 (13.25)	−2.434	**0.011**	0.65
Psychosis	1 (12.5)	1 (4)	0 (9.25)	−2.197	**0.031**	0.59
Affective symptom	1 (4)	0.5 (1)	0 (3)	−1.997	0.063	0.53
Apathy	7 (12)	0.5 (3)	0 (8.5)	−2.201	**0.031**	0.59

*Note:* Data were presented as median (interquartile range). Bolded values indicate *p* < 0.05.

Abbreviations: *APOE*, apolipoprotein E; CDR, Clinical Dementia Rating; HAMA, Hamilton Anxiety Scale; HAMD, Hamilton Depression Rating Scale; IQR, interquartile range; M0, baseline; M6, 6 months of follow‐up; MMSE, Mini‐Mental State Examination; MoCA, Montreal Cognitive Assessment; NPI, Neuropsychiatric Inventory.

### 3.2 Changes in NPS After Lecanemab Treatment

**FIGURE 1:**
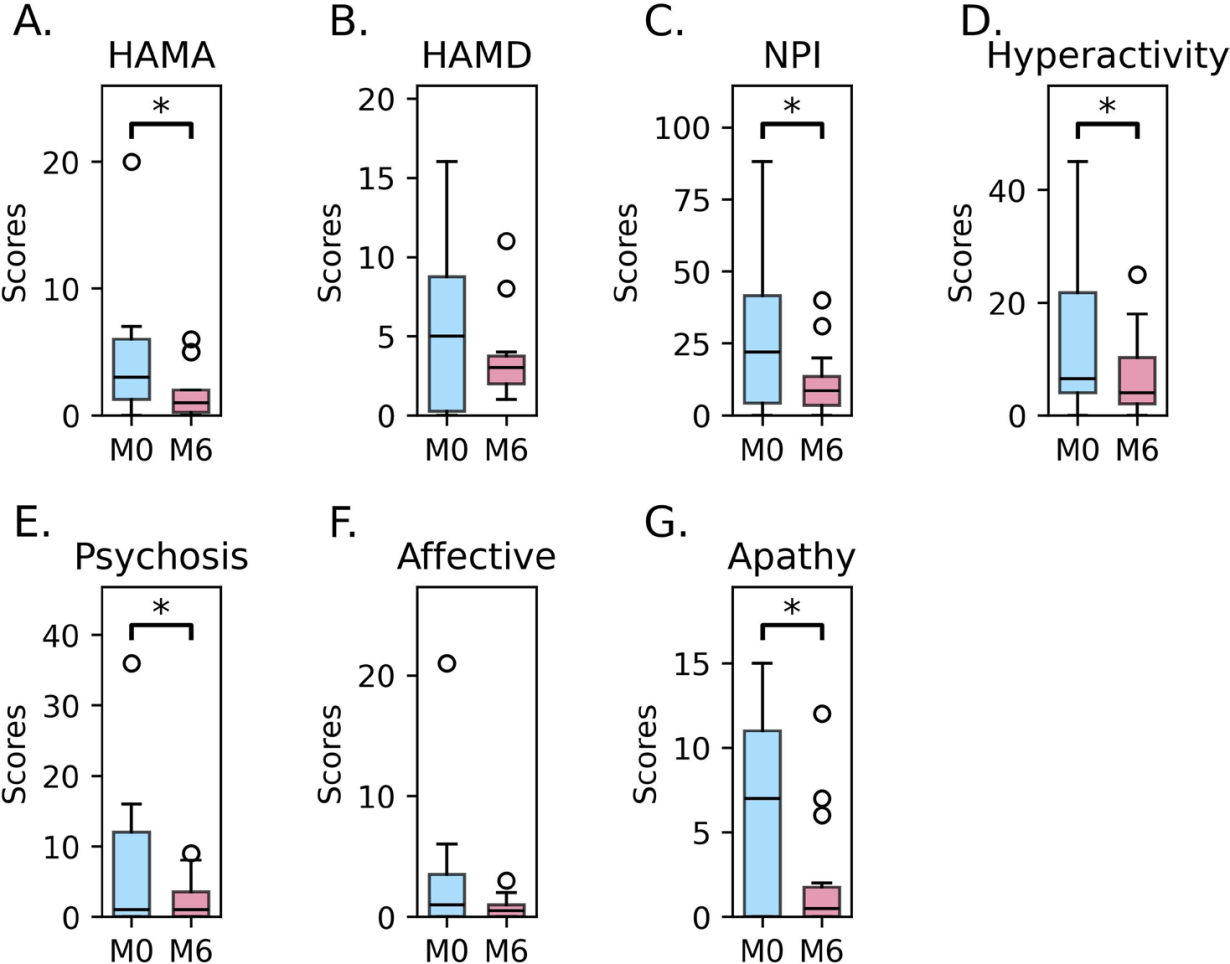
Comparison of HAMA, HAMD, total NPI score and sub‐scores at M0 and M6. HAMA (A), NPI (C), Hyperactivity (D), Psychosis (E), and Apathy (G) scores decreased at M6, while the HAMD (B) and Affective (F) scores did not change significantly. HAMD, Hamilton Depression Rating Scale; HAMA, Hamilton Anxiety Scale; NPI, Neuropsychiatric Inventory. *Indicates statistically significant. *P < 0.05.

Median HAMA scores declined from 3 [5.25] at M0 to 1 [2.75] at M6 (*p* = 0.025，Table 1 and Figure 1A), indicating a reduction in anxiety severity. HAMD scores showed a nonsignificant decrease (median [IQR]: 5 [9] vs. 3 [2], *p* = 0.250，Table 1 and Figure 1B). Total NPI scores were lower at M6 (8.5 [12.5]) than at M0 (22 [42.25], *p* = 0.005, Table 1 and Figure 1C). Among the NPI subsyndromes, hyperactivity (6.5 [21.75] vs. 4 [9.5],*p*=0.011, Table 1 and Figure 1D), psychosis (1 [12.5] vs. 1 [4], *p* = 0.031, Table 1 and Figure 1E) and apathy (7 [12] vs. 0.5 [3], *p* = 0.031,Table 1 and Figure 1G) showed significant improvement after 6 months of treatment. The affective subsymptom score exhibited a numerical decline, this change did not reach statistical significance (Table 1 and Figure 1F). No significant differences were observed between APOE ε4 carriers and noncarriers regarding MMSE, MoCA, HAMA, HAMD, total NPI, or NPI subsyndromes scores (all *p* > 0.05, Table S1).

### 3.3 Change in Aβ Burden After Lecanemab Treatment

Aβ burden was quantified using SUVr derived from ^11^C‐PIB PET images and CL values computed according to the CL Project protocol [20]. At M6, mean SUVr values were lower than at M0 in most cortical regions, including the frontal and temporal lobes (Table 2), consistent with a treatment‐associated reduction in Aβ burden. Mean (SD) global CL values decreased from 67.63 (46.44) at M0 to 35.93 (35.26) at M6 (*p* < 0.01). Longitudinal changes in Aβ burden across regional brain ROIs were comparable between APOE ε4 carriers and noncarriers (all *p* > 0.05; Tables S2 and S3).

**TABLE 2:** Mean SUVr within ROIs.

ROIs	M0	M6	M6‐M0	*z* values	*p*
Hippocampus					
L	1.29 (0.23)	1.14 (0.12)	−0.14 (0.08)	−3.296	**0.001**
R	1.3 (0.25)	1.19 (0.09)	−0.1 (0.12)	−3.296	**0.001**
Para‐Hippocampal					
L	1.16 (0.21)	1.04 (0.16)	−0.11 (0.07)	−3.296	**0.001**
R	1.16 (0.27)	1.09 (0.14)	−0.11 (0.1)	−3.233	**0.001**
Amygdala					
L	1.26 (0.23)	1.13 (0.18)	−0.15 (0.12)	−3.107	**0.002**
R	1.32 (0.2)	1.18 (0.24)	−0.19 (0.12)	−3.17	**0.002**
Thalamus					
L	1.57 (0.23)	1.21 (0.18)	−0.35 (0.23)	−3.296	**0.001**
R	1.47 (0.27)	1.13 (0.22)	−0.35 (0.21)	−3.107	**0.002**
Olfactory tubercle					
L	1.62 (0.28)	1.33 (0.28)	−0.26 (0.14)	−3.296	**0.001**
R	1.53 (0.41)	1.24 (0.34)	−0.25 (0.16)	−3.233	**0.001**
Anterior Cingulate cortex					
L	1.9 (0.45)	1.55 (0.38)	−0.33 (0.2)	−3.296	**0.001**
R	1.89 (0.47)	1.51 (0.32)	−0.37 (0.14)	−3.296	**0.001**
Middle Cingulate cortex					
L	2 (0.62)	1.55 (0.51)	−0.38 (0.16)	−3.296	**0.001**
R	1.95 (0.54)	1.47 (0.41)	−0.37 (0.17)	−3.233	**0.001**
Posterior Cingulate cortex					
L	1.96 (0.53)	1.72 (0.41)	−0.2 (0.12)	−3.296	**0.001**
R	1.85 (0.44)	1.75 (0.47)	−0.12 (0.24)	−2.856	**0.004**
Superior Frontal Gyrus					
L	1.51 (0.48)	1.31 (0.38)	−0.21 (0.18)	−3.296	**0.001**
R	1.59 (0.4)	1.39 (0.25)	−0.2 (0.1)	−3.107	**0.002**
Medial OFC					
L	1.84 (0.59)	1.52 (0.52)	−0.25 (0.17)	−3.233	**0.001**
R	1.84 (0.45)	1.5 (0.47)	−0.22 (0.15)	−3.107	**0.002**
Middle Frontal gyrus					
L	1.6 (0.44)	1.33 (0.47)	−0.24 (0.2)	−3.296	**0.001**
R	1.7 (0.46)	1.38 (0.36)	−0.23 (0.08)	−3.170	**0.002**
Anterior OFC					
L	1.7 (0.45)	1.39 (0.53)	−0.23 (0.17)	−3.296	**0.001**
R	1.75 (0.58)	1.44 (0.66)	−0.21 (0.19)	−2.982	**0.003**
Opercular part of IFG					
L	1.52 (0.35)	1.36 (0.24)	−0.19 (0.27)	−3.296	**0.001**
R	1.53 (0.37)	1.33 (0.22)	−0.22 (0.15)	−3.170	**0.002**
Triangular part of IFG					
L	1.56 (0.36)	1.33 (0.37)	−0.26 (0.24)	−3.296	**0.001**
R	1.62 (0.42)	1.38 (0.28)	−0.21 (0.13)	−3.107	**0.002**
Posterior OFC					
L	1.55 (0.39)	1.24 (0.33)	−0.24 (0.2)	−3.296	**0.001**
R	1.46 (0.45)	1.21 (0.35)	−0.22 (0.15)	−3.107	**0.002**

*Note:* Data were presented as median (interquartile range). Bolded values indicate *p* < 0.05; *p* values were FDR corrected.

Abbreviations: IFG, inferior frontal gyrus; L, left; M0, baseline; M6, 6 months of follow‐up; OFC, orbitofrontal cortex; R, right; ROIs, regions of interest; SUVr, standardized uptake value ratio.

### 3.4 Relationship Between Aβ Clearance and NPS Improvement

To investigate the relationship between Aβ clearance and NPS improvement, we focused on anatomically and functionally relevant ROIs, including the thalamus, hippocampus, para‐hippocampal gyrus, amygdala, frontal lobe, olfactory tubercle, and cingulate cortex. Mean SUVr values in these regions were significantly lower at M6 than at M0 (all *p* < 0.05, FDR‐corrected, Table 2).

**FIGURE 2:**
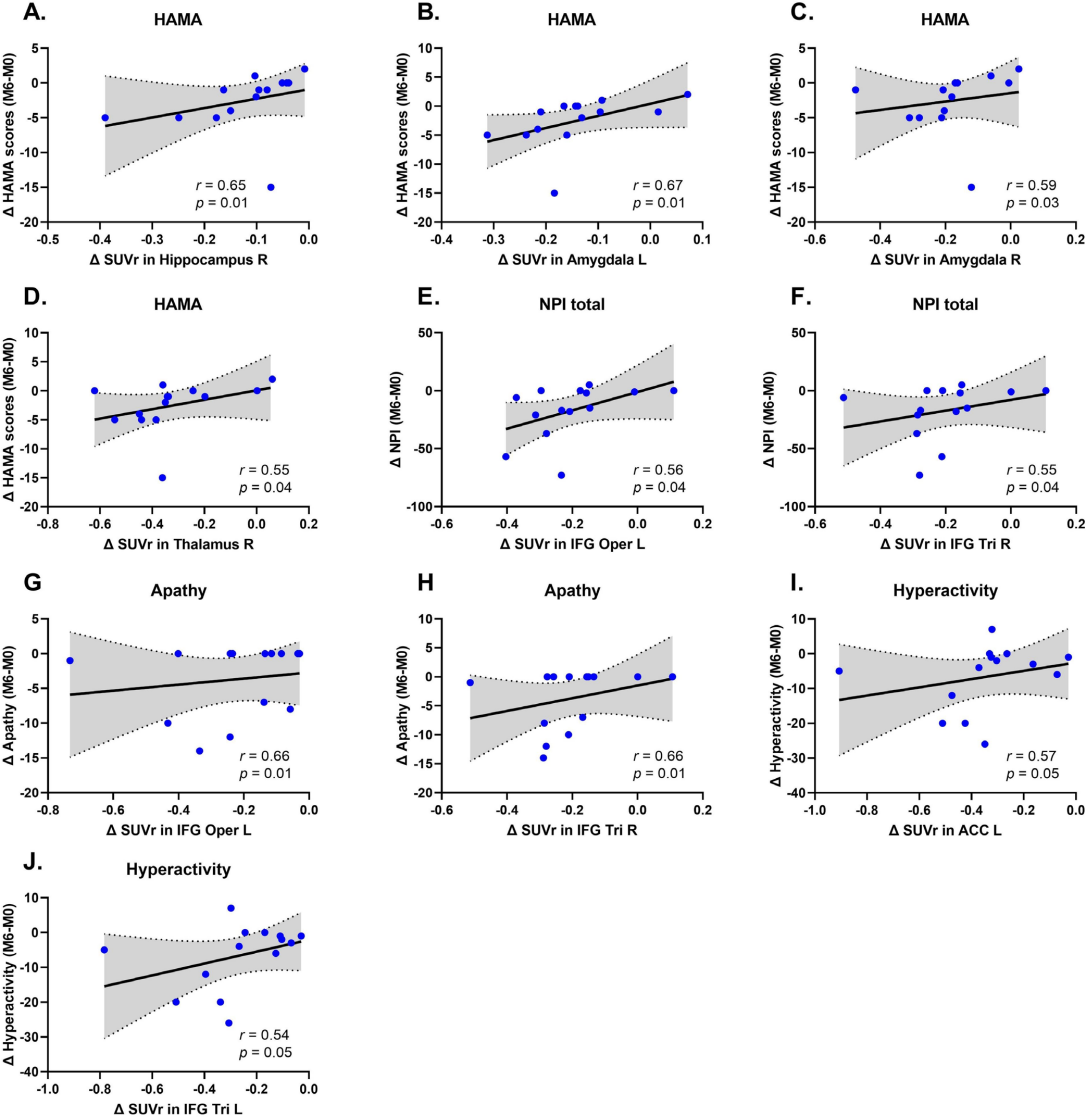
Correlation between regional Aβ clearance and NPS symptom improvement. (A–D) Decreased HAMA scores correlated with greater Aβ clearance in the right hippocampus, bilateral amygdala, and right thalamus. (E–H) Decreased total NPI and apathy scores correlated with greater Aβ clearance in IFG. (I, J) Decreased hyperactivity scores correlated with Aβ load reduction in the left ACC and left IFG. ACC, anterior cingulate cortex; IFG, inferior frontal gyrus; L, left; R, right; Δ, difference between measurements at M6 and M0.

Correlation analyses suggested that greater reductions in Aβ burden were associated with larger decreases in anxiety (ΔHAMA) in the right hippocampus (*r* = 0.65, *p* = 0.01), left amygdala (*r* = 0.67, *p* = 0.01), right amygdala (*r* = 0.59, *p* = 0.03), and right thalamus (*r* = 0.55, *p* = 0.04, Figure 2A–D). Similarly, a greater decline in total NPI score was associated with greater Aβ clearance in the left inferior frontal gyrus (IFG) (*r* = 0.56, *p* = 0.04, Figure 2E) and right IFG (*r* = 0.55, *p* = 0.04, Figure 2F). A greater reduction in apathy scores was associated with Aβ reduction in the left and right IFG (both *r* = 0.66, *p* = 0.01, Figure 2G,H), while lower hyperactivity scores were associated with reduced Aβ burden in the left anterior cingulate gyrus (ACC, *r* = 0.57, *p* = 0.05, Figure 2I) and left IFG (*r* = 0.54, *p* = 0.05, Figure 2J). No significant associations were observed between regional Aβ clearance and changes in psychosis or affective symptoms, or between CL change and changes in cognitive or NPS (all *p* > 0.05, Table S4).

### 3.5 Cortical Volume and Morphological Metric Changes After Lecanemab Treatment

**FIGURE 3:**
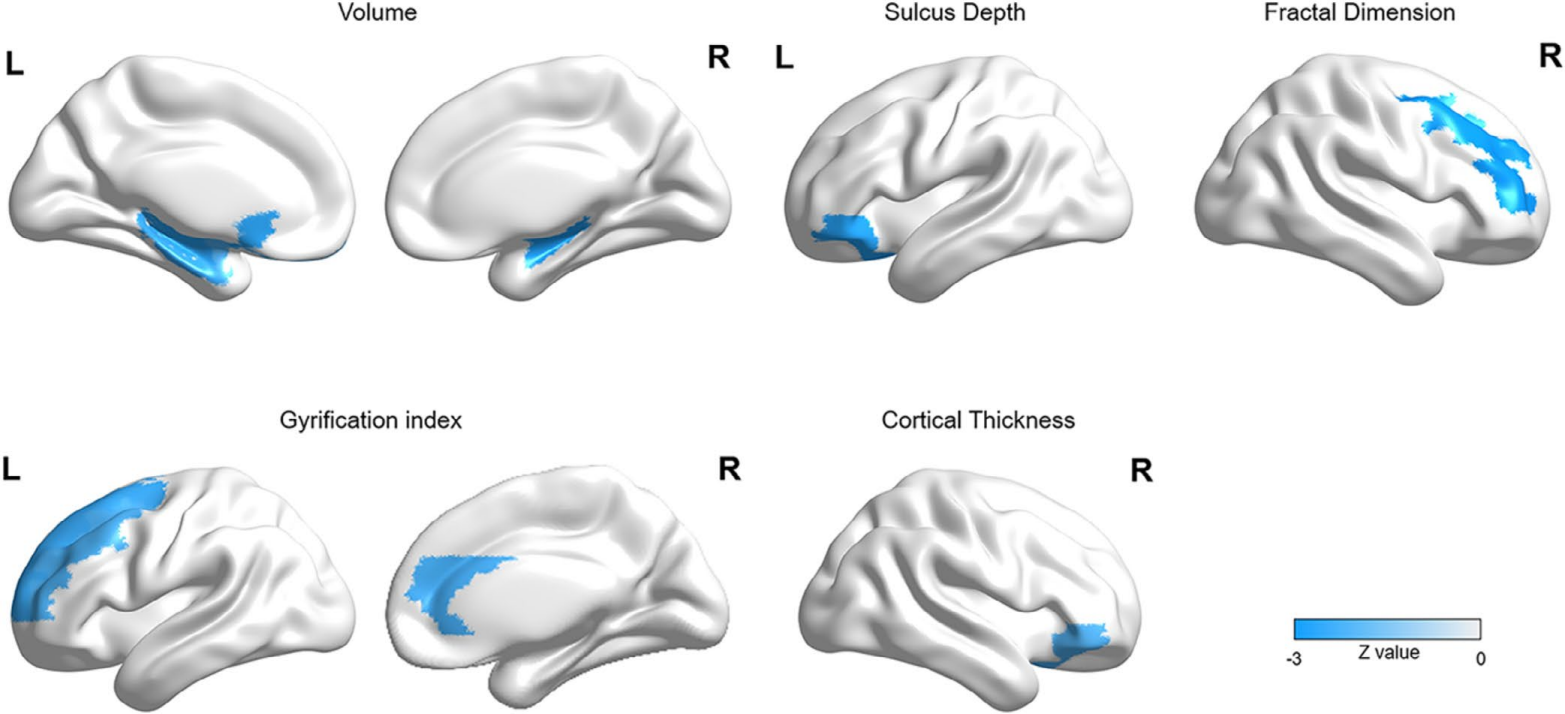
Cortical volume and surface metrics decreased in regional ROIs after 6 months of lecanemab treatment (uncorrected). L, left; R, right.

Neuro‐morphometric analyses revealed region‐specific structural changes following lecanemab treatment. At M6, volumes of the bilateral hippocampus, left para‐hippocampal gyrus, left amygdala, left olfactory tubercle and left superior frontal gyrus were lower than at M0 (all *p* < 0.05, uncorrected, Figure 3; Table S5). Surface‐based morphometric analysis further demonstrated changes in cortical thickness, gyrification index, sulcal depth and fractal dimension (FD) (Figure 3). Cortical thickness in the right inferior frontal gyrus decreased after 6 months of lecanemab treatment (*p* < 0.05, uncorrected). FD increased in the left middle cingulate but decreased in the right frontal middle gyrus after 6 months of treatment (*p* < 0.05, uncorrected). At M6, sulcal depth of left orbital part of the IFG was reduced compared to M0 (*p* < 0.05, uncorrected). Additionally, the gyrification index declined in the right anterior cingulate, left middle frontal gyrus, and left superior frontal gyrus after treatment (all *p* < 0.05, uncorrected, Figure 3).

### 3.6 Relationship Between MRI Metric Changes and NPS Improvement

**FIGURE 4:**
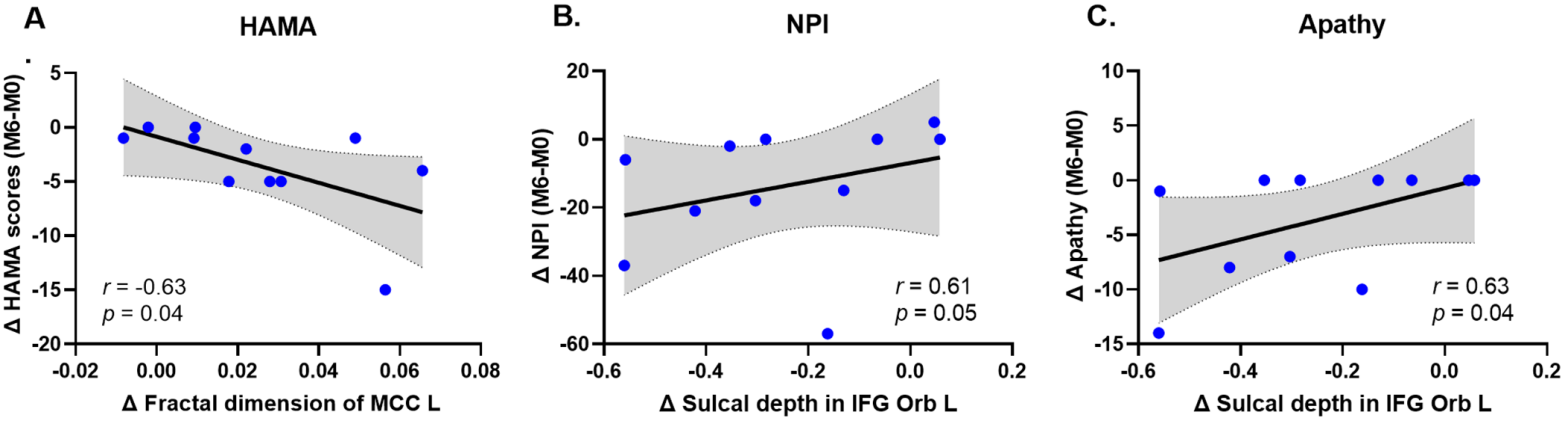
Correlation between cortical surface metrics and NPS changes. (A) Reduction in HAMA scores correlated inversely with decreased fractal dimension in left MCC; (B, C) Reduction in total NPI score and apathy scores correlated with decreased sulcal depth in left IFG orb. IFG orb, Inferior frontal gyrus pars orbitalis; L, left; MCC, Middle cingulate cortex; R, right; Δ, difference between measurements at M6 and M0.

Increased FD in the left middle cingulate cortex was associated with greater reduction in HAMA scores (*r* =−0.63, *p* = 0.04, Figure 4A, Table S6). Decreased sulcal depth in the left orbital part of the IFG was associated with greater decline in total NPI score (*r* = 0.61, *p* = 0.05, Figure 4B) and apathy scores (*r* = 0.63, *p* = 0.04, Figure 4C). HAMD scores and NPI sub‐scores for hyperactivity, psychosis, and affective symptoms showed no significant correlations with any MRI‐derived metric (all *p* > 0.05).

## 4 Discussion

In this study, we evaluated the association of 6 months of lecanemab treatment on Aβ clearance and NPS change in patients with early‐stage AD. Our analysis showed that cerebral Aβ burden decreased over the 6‐month lecanemab treatment period, which was accompanied by improvements in HAMA scores, total NPI score, and NPI sub‐scores for psychosis, hyperactivity, and apathy. We further observed that the improvement in these neuropsychiatric symptoms was correlated with reduced Aβ deposition in target cortical regions. Early changes in FD and sulcal depth in certain regions were observed, and these changes were associated with the observed neuropsychiatric improvements.

Recent retrospective analyses of phase II/III trials have shown that less cognitive decline was associated with the magnitude of amyloid removal, quantified by CL [22]. In a longitudinal follow‐up study involving patients with MCI, 12 months of lecanemab treatment resulted in significant improvements in CSF biomarkers, whereas MMSE, MoCA, Clinical Dementia Rating Scale‐Sum of Boxes (CDR‐SB), or Alzheimer's Disease Assessment Scale‐Cognitive Subscale (ADAS‐Cog) scores showed no measurable deterioration [23]. Consistent with these observations, our data suggest that 6 months of lecanemab treatment was associated with reduced Aβ burden, quantified by SUVr and CL, across multiple brain regions. MMSE and MoCA scores remained stable over time. These findings suggest that lecanemab may attenuate cognitive decline or facilitate the maintenance of cognitive stability by reducing Aβ deposition. Long‐term follow‐up is currently underway to assess the durability of these effects.

While the connection between NPS and Aβ burden in AD is documented in several studies [11, 24, 25, 26, 27, 28], there is still very little clinical data showing how anti‐Aβ treatments affect NPS [6]. In our study, 6 months of lecanemab treatment was associated with a lower total NPI score, and reductions in Aβ burden were associated with improvement in total NPI score. These findings are consistent with an association between NPS and cerebral Aβ deposition. We also examined whether regional changes in Aβ deposition were associated with total NPI score, and found that lower total NPI score was associated with greater Aβ clearance in the IFG. Although a previous MRI study highlighted the critical role of the IFG in affective dysregulation in AD [29], the relation between IFG Aβ deposition and NPS has not been well characterized. The potential contribution of IFG Aβ deposition to NPS therefore needs further study. Given the intrinsic heterogeneity of NPS, we also examined NPI sub‐scores (hyperactivity, psychosis, affective symptoms, apathy) and symptom‐specific rating scales such as the HAMA and HAMD. These analyses indicate that lecanemab treatment may be associated with improvement in several NPS domains, including anxiety, apathy, psychosis, and hyperactivity.

Longitudinal cohort studies have established that Aβ pathology may contribute to the development of anxiety [11, 27, 28]. Previous PET investigations have demonstrated that patients with anxiety exhibit higher Aβ deposition in the precuneus‐posterior cingulate, frontal lobe, parietal lobe, ACC, thalamus, and amygdala [24]. Furthermore, a systematic review indicated that the anterior and posterior cingulate cortices, entorhinal cortex, para‐hippocampal gyrus, insular cortex, and amygdala represent the most frequently implicated brain regions in anxiety‐related pathophysiology [30]. Partially consistent with these findings, our correlation analyses showed that more pronounced reductions in Aβ burden within the right hippocampus, bilateral amygdala, and right thalamus were associated with greater improvements in anxiety scores. Although the underlying mechanisms remain unclear, these findings suggest that the hippocampus, bilateral amygdala, and thalamus may be the focus regions for future research.

Multiple studies have demonstrated that apathy in AD correlates with Aβ deposition in the bilateral frontal lobe, prefrontal cortex, medial and orbitofrontal areas, insula, and right ACC [31, 32, 33]. Our neuroimaging findings extend previous observations and suggest that improvement in apathy scores may be associated with reduced Aβ burden in the left IFG during lecanemab treatment. These preliminary data support a possible role of the prefrontal cortex, particularly the IFG, in the pathophysiology of apathy.

The hyperactivity syndrome includes agitation, disinhibition, irritability, euphoria, and aberrant motor behavior and has been associated with increased functional connectivity in the ACC and right insular regions of the salience network [30]. Although the relationship between hyperactivity and Aβ deposition remains under‐characterized, we observed that lower hyperactivity scores were associated with decreased Aβ burden in the left ACC and left IFG. These findings may indicate that hyperactivity is related to Aβ deposition in these brain regions.

A previous study found that Aβ pathology may contribute to the development of apathy and anxiety, largely independent of cognitive changes [26]. The key pathophysiological mechanisms driving the various NPS in AD have been linked to neurotransmitter systems, including serotonin (5‐HT), dopamine (DA), and norepinephrine (NE) [7]. Preclinical evidence has demonstrated that amyloid plaques induce deficits within neurotransmitter systems that are associated with behavioral abnormalities [18]. Furthermore, soluble Aβ can also decrease 5‐HT levels in brain tissue, particularly in the prefrontal cortex, which may contribute to emotional symptoms [34]. In our study, cognitive function remained stable, whereas lecanemab treatment was associated with significant improvement in multiple NPS domains. This pattern raises the possibility that changes in NPS may not fully mirror changes in global cognitive scores. However, the underlying mechanisms remain uncertain. Further investigations should explore the effects of anti‐Aβ therapies on NPS using imaging or biomarker measures of neurotransmission.

The relationship between depression and Aβ pathology in early‐stage AD is not very clear. While depression has been reported to correlate with frontal Aβ deposition in MCI [35], other studies have observed weak or negligible associations between depression and Aβ pathology [36, 37]. In our study, HAMD scores and affective NPI sub‐scores for anxiety and depression showed numerical improvement, but the changes were not statistically significant. These results underscore the necessity for further investigation into the relationship between depression and cerebral Aβ deposition in early‐stage AD. However, the lack of statistically significant findings may be attributable to several factors, including the limited sample size, short follow‐up, or other confounding variables, including psychosocial and environmental factors and comorbidities [37].

Brain atrophy and diminished cortical complexity are typical morphological alterations observed during the natural course of AD [38]. However, whether anti‐Aβ therapy can modify these structural trajectories remains unclear. In this study, we observed post‐treatment changes in several morphometric parameters, including decreased gray matter volume, gyrification index, and sulcal depth in specific brain regions; although these findings did not survive FDR correction. In addition, increased FD in the left middle cingulate cortex was associated with reduced HAMA scores, and decreased sulcal depth in the left orbital part of the IFG was associated with lower total NPI and apathy scores. These results should be interpreted with caution because the structural findings did not pass multiple‐comparison correction. Larger studies are required to determine whether morphological changes could serve as reliable imaging biomarkers for evaluating the therapeutic efficacy of anti‐Aβ in AD.

Several limitations should be acknowledged. The absence of an untreated control group with repeat PET scans, largely due to recruitment challenges, limits our ability to separate lecanemab‐associated changes from natural disease progression. Combined with the small sample size, this design may have reduced statistical power and increased the risk of type II errors. Participants also remained on stable doses of cholinesterase inhibitors or NMDA receptor antagonists throughout the study. Although the contribution of these background medications to NPS changes cannot be excluded, their efficacy for neuropsychiatric symptoms is generally modest or limited [39]. Accordingly, background therapy alone is unlikely to fully explain the observed 6‐month changes. However, this possibility cannot be ruled out. Larger studies with stratified analyses by concomitant medication are needed to better isolate the association attributable to lecanemab.

The 6‐month follow‐up was insufficient to assess long‐term efficacy. Ongoing follow‐up will be essential to determine whether anti‐Aβ therapy confers sustained improvements in NPS scores. Finally, the study did not include blood or CSF biomarkers relevant to NPS. Future research should incorporate neuroimaging studies of neurotransmitters to further explore the underlying mechanisms. Overall, these limitations underscore the need for larger, placebo‐controlled trials to validate and extend the present observations.

## 5 Conclusion

In summary, this exploratory study provides preliminary evidence that 6 months of lecanemab treatment may be associated with improvements in NPS, particularly anxiety, apathy, and hyperactivity. These clinical improvements were correlated with regional Aβ burden reduction. These findings are consistent with the hypothesis that anti‐Aβ therapy might improve NPS through amyloid clearance in early‐stage AD. Larger controlled trials with extended follow‐up durations are needed to confirm whether these associations are reproducible.

## Author Contributions

Yan Chang, Hua Li, Hairong Qian, and Ruimin Wang were responsible for the study concept and design. Yan Chang, Hua Li, and Xiaojie Liu were responsible for drafting the original report which was reviewed and revised by all coauthors (Yan Chang, Hua Li, Xiaojie Liu, Xuemei Li, Jiajin Liu, Jingbin Song, Huaping Fu, Xiaodan Xu, Yuan Wang, Qianyao Wang, Na Ren, Jilin Chen, Xin Deng, Xue Zhang, Luofei Zuo, Bo Zhou, Xuan Sun, Zihan Li, Yuanyan Cao, Runze Wu, Jianjun Jia, Hairong Qian, and Ruimin Wang). Jiajin Liu, Xiaodan Xu, Yuan Wang, and Jingbin Song were responsible for the acquisition of the PET/MR and PET/CT scan. Huaping Fu was responsible for providing the PET tracer for amyloid and tau imaging. Yan Chang, Hua Li, Xiaojie Liu, Yuanyan Cao, and Runze Wu were responsible for all neuroimaging analyses and statistical analyses. Xuemei Li, Qianyao Wang, Na Ren, Jilin Chen, Xin Deng, Xue Zhang, Luofei Zuo, Bo Zhou, Xuan Sun, and Zihan Li were responsible for the acquisition of the clinical data. Yan Chang, Hua Li, Xiaojie Liu, Yuanyan Cao, Runze Wu, Jianjun Jia, Hairong Qian, and Ruimin Wang were responsible for the interpretation of data for the work. All authors have read and approved the final version of the manuscript and agree with the order of authorship.

## Funding

This study was supported by the National Natural Science Foundation of China (82371999); the Ministry of Science and Technology of the People's Republic of China (2021ZD0201804); Health Special Research Projects (24BJZ14); and Novel Medical Technologies and Innovative Services of Chinese PLA General Hospital.

## Ethics Statement

The study was conducted in accordance with the Declaration of Helsinki. The study was approved by the Human Ethics Committee of Chinese PLA General Hospital (S2025‐052‐01).

## Consent

Written informed consent was obtained from all participants.

## Conflicts of Interest

The authors declare no conflicts of interest.

## Supporting information


**Table S1:** Comparison of cognitive and neuropsychiatric features between APOE ε4 carriers and noncarriers.
**Table S2:** Comparison of regional brain SUVr values between APOE ε4 carriers and noncarriers.
**Table S3:** Comparison of global CL values between APOE ε4 carriers and noncarriers.
**Table S4:** Association between neuropsychiatric symptoms assessment and SUVr.
**Table S5:** Comparison of volume and surface measurements at M0 and M6.
**Table S6:** *p* values of Spearman correlation analysis between neuropsychiatric assessment changes and MR image measurement changes.

## Data Availability

The datasets generated and analyzed during this study are not publicly available due to institutional data protection regulations. However, de‐identified regional summary measures and analyzed PET/MRI metrics can be made available from the corresponding author upon reasonable request for the purposes of transparency and reproducibility.
