# Supplementary material for: Lecanemab Reduces Neuropsychiatric Symptoms and Related Regional Brain Amyloid Load in Early Alzheimer's Disease: A Preliminary Prospective Study
Source: CNS Neurosci Ther. 2026 Jun 11;32(6):e70974. doi: 10.1002/cns.70974 (PMC13259964; doi:10.1002/cns.70974)
Supplement: Supplementary file 1 — Table S1: Comparison of cognitive and neuropsychiatric features between APOE ε4 carriers and noncarriers. Table S2: Comparison of regional brain SUVr values between APOE ε4 carriers and noncarriers. Table S3: Comparison of global CL values between APOE ε4 carriers and noncarriers. Table S4: Association between neuropsychiatric symptoms assessment and SUVr. Table S5: Comparison of volume and surface measurements at M0 and M6. Table S6: p values of Spearman correlation analysis between neuropsychiatric assessment changes and MR image measurement changes. [file CNS-32-e70974-s001.docx]

**PET image processing method**

All ^11^C-PIB PET images were first co-registered to each subject’s corresponding high-resolution T1MR images using FMRIB Software Library (FSL) to eliminate possible head movement during scanning. The co-registered PET images were spatially normalized to the Montreal Neurological Institute (MNI) standard space by applying the identical deformation field that had been computed when transforming the individual T1MR images to the MNI 152 template. The T1MR images underwent a standardized preprocessing pipeline implemented in FSL. Non-brain tissue was removed using the Brain Extraction Tool (BET), followed by spatial normalization to the MNI-152 template via affine transformation. After normalization, a set of a priori regions of interest (ROIs) was defined on the MNI template using the Automated Anatomical Labeling (AAL) atlas. The selected ROIs included thalamus, hippocampus, parahippocampal gyrus, amygdala, frontal lobe, olfactory tubercle and cingulate cortex. Mean SUVr values were extracted from each ROI for every subject, providing quantitative measures of amyloid burden for subsequent statistical analyses. SUVr values were computed for cortical gray matter using cerebellar gray matter as the reference region. CL (Centiloid) values were calculated for every participant: the 50-70 min post-injection frames were processed using the Level-2 Centiloid pipeline, and global cortical CL values were derived with the standard AD-CTX target and cerebellar reference VOIs. The observed decline in both SUVr and CL metrics provided convergent evidence of effective amyloid clearance, further validating the biological efficacy of the therapeutic intervention.

**MR image processing method**

Following the image processing procedures described above, the MR images were obtained in MNI standard space. Gray matter volumes of ROIs were extracted from standardized T1MR images. A set of cortical surface parameters were extracted from processed surface images which were processed in CAT toolbox. The cortical surface measurements included gyrification index, fractal dimension, sulcal depth and cortical thickness. Mean cortical volume and surface measurements of selected ROIs were extracted for further analysis, which included thalamus, hippocampus, para-hippocampal gyrus, amygdala, frontal lobe, olfactory tubercle and cingulate gyrus.

**Centiloid changes after lecanemab treatment**

The image processing workflow employed in this study was rigorously validated against the standard ^11^C-PIB dataset released by the GAAIN CL project. We derived a study-specific transformation equation that converts regional SUVr into CL:

CL=100×(SUVR-1.007)÷(2.03-1.007)

Our pipeline demonstrated excellent agreement with the reference CL standard: Pearson’s r = 0.983, intercept = 0.951, and R2 = 0.986, all within the recommended thresholds.

Table S1. Comparison of cognitive and neuropsychiatric features between APOE ε4 carriers and non-carriers.

|  | APOE ε4 carrier | | | APOEε4 non-carrier | | | *p* values | *z* values |
| --- | --- | --- | --- | --- | --- | --- | --- | --- |
|  | **M0** | **M6** | **M6-M0** | **M0** | **M6** | **M6-M0** |  |  |
| HAMD | 6.00 (3.75) | 3.00 (0.75) | -3.00 (2.25) | 1.50 (8.25) | 3.00 (2.00) | 1.50 (4.00) | 0.27 | -1.16 |
| HAMA | 6.00 (0.00) | 1.50 (1.00) | -4.50 (3.25) | 1.50 (2.50) | 0.50 (2.75) | -0.50 (1.50) | 0.13 | -1.55 |
| NPI | 3.00 (3.00) | 5.50 (10.25) | 4.00 (6.50) | 1.00 (8.50) | 7.50 (13.75) | 1.50 (12.00) | 0.65 | 0.52 |
| MMSE | 25.50 (4.75) | 24.50 (4.00) | -0.50 (1.75) | 23.50 (2.75) | 23.00 (4.25) | 0.00 (2.75) | 0.79 | 0.32 |
| MoCA | 20.50 (6.00) | 19.50 (4.00) | 1.50 (3.25) | 19.50 (4.75) | 20.50 (2.75) | 0.50 (2.25) | 0.85 | 0.26 |
| Hyperactivity | 4.50 (4.75) | 3.00 (5.00) | -1.50 (3.25) | 18.00 (24.50) | 4.00 (11.75) | -9.00 (17.50) | 0.08 | 1.81 |
| Psychosis | 0.00 (1.50) | 0.50 (1.75) | 0.00 (0.00) | 11.00 (12.50) | 1.00 (4.00) | -6.50 (10.25) | 0.06 | 1.81 |
| Affective symptoms | 1.00 (0.75) | 0.50 (1.00) | 0.00 (1.50) | 0.50 (4.00) | 0.50 (1.25) | 0.00 (3.00) | 1.00 | 0.00 |
| Apathy | 0.00 (4.50) | 0.00 (4.50) | 0.00 (0.00) | 10.00 (5.75) | 1.00 (1.25) | -7.50 (10.50) | 0.06 | 1.74 |

Table S2. Comparison of regional brain SUVr values between APOE ε4 carriers and non-carriers.

| ROIs | APOE ε4 carrier | | | APOE ε4 non-carrier | | | *p* values | *z* values |
| --- | --- | --- | --- | --- | --- | --- | --- | --- |
|  | **M0** | **M6** | **M6-M0** | **M0** | **M6** | **M6-M0** |  |  |
| Hippocampus L | 1.35 (0.14) | 1.19 (0.10) | -0.15 (0.07) | 1.24 (0.18) | 1.12 (0.11) | -0.13 (0.06) | 0.23 | -1.29 |
| Hippocampus R | 1.34 (0.15) | 1.19 (0.02) | -0.16 (0.12) | 1.27 (0.16) | 1.18 (0.13) | -0.08 (0.05) | 0.08 | -1.81 |
| ParaHippocampal L | 1.20 (0.12) | 1.06 (0.05) | -0.15 (0.04) | 1.08 (0.16) | 0.97 (0.16) | -0.11 (0.04) | 0.11 | -1.68 |
| ParaHippocampal R | 1.20 (0.18) | 1.09 (0.06) | -0.12 (0.07) | 1.11 (0.27) | 1.06 (0.15) | -0.10 (0.06) | 0.28 | -1.16 |
| Amygdala L | 1.26 (0.09) | 1.07 (0.12) | -0.19 (0.08) | 1.24 (0.23) | 1.18 (0.17) | -0.14 (0.07) | 0.18 | -1.42 |
| Amygdala R | 1.44 (0.10) | 1.18 (0.21) | -0.21 (0.06) | 1.30 (0.13) | 1.18 (0.16) | -0.14 (0.14) | 0.06 | -1.94 |
| Thalamus L | 1.65 (0.11) | 1.21 (0.10) | -0.40 (0.15) | 1.47 (0.20) | 1.20 (0.18) | -0.27 (0.19) | 0.06 | -1.94 |
| Thalamus R | 1.55 (0.17) | 1.16 (0.18) | -0.41 (0.10) | 1.37 (0.22) | 1.13 (0.17) | -0.35 (0.21) | 0.23 | -1.29 |
| Olfactory tubercle L | 1.67 (0.22) | 1.39 (0.24) | -0.25 (0.12) | 1.58 (0.28) | 1.29 (0.29) | -0.29 (0.16) | 0.85 | -0.26 |
| Olfactory tubercle R | 1.59 (0.15) | 1.26 (0.15) | -0.27 (0.10) | 1.32 (0.39) | 1.09 (0.35) | -0.24 (0.14) | 0.41 | -0.90 |
| Anterior Cingulate cortex L | 1.96 (0.20) | 1.62 (0.14) | -0.33 (0.04) | 1.82 (0.49) | 1.44 (0.51) | -0.31 (0.30) | 0.57 | -0.65 |
| Anterior Cingulate cortex R | 1.97 (0.24) | 1.57 (0.15) | -0.41 (0.08) | 1.80 (0.58) | 1.48 (0.42) | -0.32 (0.14) | 0.11 | -1.68 |
| Middle Cingulate cortex L | 2.18 (0.23) | 1.64 (0.26) | -0.39 (0.12) | 1.88 (0.63) | 1.45 (0.42) | -0.37 (0.21) | 0.34 | -1.03 |
| Middle Cingulate cortex R | 2.10 (0.30) | 1.56 (0.29) | -0.41 (0.10) | 1.82 (0.64) | 1.43 (0.37) | -0.36 (0.24) | 0.23 | -1.29 |
| Posterior Cingulate cortex L | 2.01 (0.43) | 1.72 (0.10) | -0.24 (0.10) | 1.90 (0.40) | 1.69 (0.46) | -0.16 (0.17) | 0.08 | -1.81 |
| Posterior Cingulate cortex R | 1.97 (0.51) | 1.76 (0.30) | -0.22 (0.07) | 1.80 (0.38) | 1.68 (0.40) | -0.04 (0.15) | 0.14 | -1.55 |
| Superior Frontal Gyrus L | 1.55 (0.24) | 1.27 (0.19) | -0.23 (0.07) | 1.50 (0.50) | 1.32 (0.41) | -0.18 (0.16) | 0.28 | -1.16 |
| Superior Frontal Gyrus R | 1.61 (0.21) | 1.36 (0.08) | -0.23 (0.08) | 1.58 (0.53) | 1.41 (0.29) | -0.18 (0.15) | 0.23 | -1.29 |
| Medial OFC L | 1.89 (0.23) | 1.54 (0.24) | -0.28 (0.05) | 1.75 (0.53) | 1.47 (0.59) | -0.24 (0.29) | 0.49 | -0.77 |
| Medial OFC R | 1.89 (0.22) | 1.50 (0.24) | -0.27 (0.06) | 1.76 (0.57) | 1.52 (0.57) | -0.15 (0.10) | 0.03 | -2.19 |
| Middle Frontal gyrus L | 1.56 (0.26) | 1.28 (0.22) | -0.23 (0.11) | 1.60 (0.48) | 1.36 (0.52) | -0.24 (0.21) | 0.85 | -0.26 |
| Middle Frontal gyrus R | 1.65 (0.30) | 1.35 (0.09) | -0.26 (0.09) | 1.71 (0.57) | 1.48 (0.41) | -0.20 (0.12) | 0.11 | -1.68 |
| Anterior OFC L | 1.74 (0.12) | 1.41 (0.39) | -0.23 (0.10) | 1.62 (0.50) | 1.30 (0.52) | -0.25 (0.26) | 0.85 | -0.26 |
| Anterior OFC R | 1.81 (0.15) | 1.46 (0.36) | -0.25 (0.10) | 1.52 (0.57) | 1.37 (0.62) | -0.14 (0.22) | 0.06 | -1.94 |
| Opercular part of IFG L | 1.55 (0.13) | 1.36 (0.10) | -0.18 (0.12) | 1.50 (0.43) | 1.33 (0.28) | -0.19 (0.27) | 0.85 | 0.26 |
| Opercular part of IFG R | 1.55 (0.29) | 1.33 (0.16) | -0.20 (0.12) | 1.53 (0.43) | 1.29 (0.25) | -0.22 (0.18) | 0.85 | -0.26 |
| Triangular part of IFG L | 1.60 (0.19) | 1.31 (0.19) | -0.22 (0.17) | 1.50 (0.43) | 1.33 (0.38) | -0.28 (0.24) | 0.95 | 0.13 |
| Triangular part of IFG R | 1.59 (0.31) | 1.37 (0.09) | -0.23 (0.10) | 1.64 (0.54) | 1.41 (0.33) | -0.19 (0.18) | 0.66 | -0.52 |
| Posterior OFC L | 1.55 (0.34) | 1.24 (0.17) | -0.24 (0.07) | 1.55 (0.40) | 1.27 (0.41) | -0.26 (0.22) | 0.85 | -0.26 |
| Posterior OFC R | 1.47 (0.33) | 1.21 (0.04) | -0.23 (0.13) | 1.42 (0.52) | 1.18 (0.40) | -0.21 (0.12) | 0.57 | -0.65 |

Table S3. Comparison of global CL values between APOE ε4 carriers and non-carriers.

|  | APOE ε4 carrier | | | APOE ε4 non-carrier | | | *p* values | z values |
| --- | --- | --- | --- | --- | --- | --- | --- | --- |
|  | **M0** | **M6** | **M6-M0** | **M0** | **M6** | **M6-M0** |  |  |
| CL | 67.26 (34.43) | 35.64 (13.85) | -31.63 (12.43) | 68.28 (52.37) | 36.03 (42.16) | -27.88 (19.96) | 0.57 | -0.65 |

Table S4. Association between neuropsychiatric symptoms assessment and SUVr

| Neuropsychiatric symptom | ROIs | *p* value | r value |
| --- | --- | --- | --- |
| Psychosis | Hippocampus L | 0.377527912 | -0.255819916 |
| Psychosis | Hippocampus R | 0.39587623 | -0.246432029 |
| Psychosis | Amygdala L | 0.570166755 | -0.166634991 |
| Psychosis | Amygdala R | 0.262838435 | -0.321535124 |
| Psychosis | Thalamus R | 0.935679829 | -0.025816689 |
| Psychosis | Anterior Cingulate cortex L | 0.758721024 | 0.091531897 |
| Psychosis | Middle Frontal gyrus L | 0.368492734 | 0.260513859 |
| Psychosis | Opercular part of Inferior Frontal gyrus L | 0.414778624 | 0.237044142 |
| Psychosis | Opercular part of Inferior Frontal gyrus R | 0.414778624 | 0.237044142 |
| Psychosis | Triangular part of Inferior Frontal gyrus L | 0.443864353 | 0.222962312 |
| Psychosis | Triangular part of Inferior Frontal gyrus R | 0.494645748 | 0.199492595 |
| Psychosis | Posterior OFC L | 0.537216256 | 0.180716821 |
| Affective Symptoms | Hippocampus L | 0.77974618 | 0.082865233 |
| Affective Symptoms | Hippocampus R | 0.594607245 | 0.155981615 |
| Affective Symptoms | Amygdala L | 0.937144337 | 0.024372127 |
| Affective Symptoms | Amygdala R | 0.372100122 | 0.25834455 |
| Affective Symptoms | Thalamus R | 0.766244866 | -0.087739659 |
| Affective Symptoms | Anterior Cingulate cortex L | 0.242790543 | 0.333898145 |
| Affective Symptoms | Middle Frontal gyrus L | 0.192485292 | 0.370456336 |
| Affective Symptoms | Opercular part of Inferior Frontal gyrus L | 0.211729012 | 0.35583306 |
| Affective Symptoms | Opercular part of Inferior Frontal gyrus R | 0.12966848 | 0.426512229 |
| Affective Symptoms | Triangular part of Inferior Frontal gyrus L | 0.211729012 | 0.35583306 |
| Affective Symptoms | Triangular part of Inferior Frontal gyrus R | 0.160539461 | 0.397265677 |
| Affective Symptoms | Posterior OFC L | 0.192485292 | 0.370456336 |

Table S5. Comparison of volume and surface measurements at M0 and M6.

| ROIs | M0 | M6 | Z | *p* |
| --- | --- | --- | --- | --- |
| Volume of Hippocampus L | 0.465 ± 0.077 | 0.436 ± 0.061 | -2.578 | 0.01 |
| Volume of Hippocampus R | 0.463 ± 0.066 | 0.437 ± 0.058 | -2.756 | 0.006 |
| Volume of Para Hippocampus L | 0.443 ± 0.072 | 0.396 ± 0.037 | -2.401 | 0.016 |
| Volume of Amygdala L | 0.434 ± 0.121 | 0.372 ± 0.066 | -2.045 | 0.041 |
| Volume of Olfactory tubercle L | 0.507 ± 0.096 | 0.455 ± 0.066 | -2.578 | 0.01 |
| Volume of Superior Frontal Gyrus L | 0.442 ± 0.043 | 0.418 ± 0.044 | -2.045 | 0.041 |
| Sulcal depth of IFG orb L | 8.596 ± 0.964 | 8.348 ± 0.941 | -2.667 | 0.008 |
| Fractal dimension of Middle Cingulate cortex L | 2.195 ± 0.125 | 2.221 ± 0.127 | 2.667 | 0.008 |
| Fractal dimension of Middle Frontal sulcal R | 2.638 ± 0.233 | 2.571 ± 0.188 | -2.223 | 0.026 |
| Fractal dimension of Middle Frontal gyrus R | 2.697 ± 0.148 | 2.665 ± 0.146 | -2.312 | 0.021 |
| Gyrification of Anterior Cingulate cortex R | 29.68 ± 1.015 | 28.658 ± 1.079 | -2.223 | 0.026 |
| Gyrification of Middle Frontal gyrus L | 29.005 ± 1.314 | 28.435 ± 1.35 | -2.134 | 0.033 |
| Gyrification of Superior Frontal Gyrus L | 27.239 ± 1.429 | 27.699 ± 1.531 | -2.401 | 0.016 |
| Thickness of IFG orb R | 2.376 ± 0.174 | 2.3 ± 0.198 | -2.045 | 0.041 |
| Thickness of Middle Frontal gyrus L | 2.176 ± 0.211 | 2.235 ± 0.179 | 2.223 | 0.026 |
| Thickness of Superior Frontal Gyrus L | 2.465 ± 0.153 | 2.565 ± 0.117 | 2.223 | 0.026 |

Table S6. P values of Spearman correlation analysis between neuropsychiatric assessment changes and MR image measurement changes.

|  | HAMD | HAMA | Hyperactivity | Apathy | Psychosis | Affective Symptoms |
| --- | --- | --- | --- | --- | --- | --- |
| Volume of Hippocampus L | 0.885 | 0.641 | 0.984 | 0.597 | 0.913 | 0.424 |
| Volume of Hippocampus R | 0.576 | 0.554 | 0.908 | 0.314 | 0.123 | 0.272 |
| Volume of Para Hippocampus L | 0.138 | 0.185 | 0.697 | 0.971 | 0.820 | 0.749 |
| Volume of Amygdala L | 0.778 | 0.820 | 0.676 | 0.661 | 0.798 | 0.244 |
| Volume of Olfactory tubercle L | 0.604 | 0.808 | 0.214 | 0.715 | 0.342 | 0.749 |
| Volume of Superior Frontal Gyrus L | 0.239 | 0.692 | 0.188 | 0.186 | 0.889 | 0.081 |
| Sulcal depth of IFG orb L | 0.715 | 0.973 | 0.150 | ***0.034**** | 0.391 | 0.749 |
| Fractal dimension of Middle Cingulate cortex L | 0.538 | 0.380 | 0.214 | 0.536 | 0.304 | 0.795 |
| Fractal dimension of Middle Frontal sulcal R | 0.160 | ***0.041**** | 0.717 | 0.232 | 0.435 | 0.526 |
| Fractal dimension of Middle Frontal gyrus R | 0.303 | 0.672 | 0.570 | 0.982 | 0.228 | 0.891 |
| Gyrification of Anterior Cingulate cortex R | 0.614 | 0.592 | 0.122 | 0.379 | 0.510 | 0.565 |
| Gyrification of Middle Frontal gyrus L | 0.566 | 0.481 | 0.822 | 0.639 | 0.741 | 0.424 |
| Gyrification of Superior Frontal Gyrus L | 0.205 | 0.334 | 0.769 | 0.703 | 0.654 | 0.749 |
| Thickness of IFG orb R | 0.547 | 0.601 | 0.667 | 0.516 | 0.764 | 0.611 |
| Thickness of Middle Frontal gyrus L | 0.832 | 0.906 | 0.365 | 0.852 | 0.444 | 0.632 |
| Thickness of Superior Frontal Gyrus L | 0.303 | 0.241 | 0.908 | 0.876 | 0.182 | 0.842 |
| Volume of Hippocampus L | 0.458 | 0.581 | 0.068 | 0.278 | 0.296 | 0.055 |

*: *p* < 0.05
